# Guided Reflection Interventions Show No Effect on Diagnostic Accuracy in Medical Students

**DOI:** 10.3389/fpsyg.2018.02297

**Published:** 2018-11-23

**Authors:** Kathryn Ann Lambe, David Hevey, Brendan D. Kelly

**Affiliations:** ^1^School of Psychology, Trinity College Dublin, Dublin, Ireland; ^2^Trinity Centre for Health Sciences, Tallaght Hospital, Trinity College Dublin, Dublin, Ireland

**Keywords:** diagnosis, diagnostic error, diagnostic reasoning, medical education, decision-making, reflective practice, dual-process model

## Abstract

**Background:** Guided reflection interventions, in an effort to reduce diagnostic error, encourage diagnosticians to generate alternative diagnostic hypotheses and gather confirming and disconfirming evidence before making a final diagnosis. This method has been found to significantly improve diagnostic accuracy in recent studies; however, it requires a significant investment of time, and psychological theory suggests the possibility for unintended consequences owing to cognitive bias. This study compared a short and long version of a guided reflection task on improvements in diagnostic accuracy, change in diagnostic confidence, and rates of corrected diagnoses.

**Methods:** One hundred and eighty-six fourth- and fifth-year medical students diagnosed a series of fictional clinical cases, by first impressions (control condition) or by using a short or long guided reflection process, and rated their confidence in their initial diagnostic hypothesis at intervals throughout the process. In the “short” condition, participants were asked to generate two alternatives to their initial diagnostic hypothesis; in the “long” condition, six alternatives were required.

**Results:** The reflective intervention did not elicit more accurate final diagnoses than diagnosis based on first impressions only. Participants who completed a short version of the task performed similarly to those who completed a long version. Neither the short nor long form elicited significant changes in diagnostic confidence from the beginning to the end of the diagnostic process, nor did the conditions differ on the rate of corrected diagnoses.

**Conclusions:** This study finds no evidence to support the use of the guided reflection method as a diagnostic aid for novice diagnosticians, who may already use an analytical approach to diagnosis and therefore derive less benefit from this intervention than their more experienced colleagues. The results indicate some support for a shorter, less demanding version of the process, and further study is now required to identify the most efficient process to recommend to doctors.

## Introduction

Diagnostic error in the medical field is a significant concern; estimates indicate that between 10 and 15% of patients' outcomes are adversely impacted by diagnostic error (Kuhn, 2002; Graber, 2013).

While multiple factors usually contribute to diagnostic errors, cognitive factors are thought to be implicated in around three-quarters of cases (Graber et al., 2005). The dual-process model of decision-making offers an approach by which these cognitive failings may be understood (Croskerry, 2002; Redelmeier, 2005). This model posits that two systems, or modes, of thinking constantly contribute to reasoning. One system (analytical reasoning) may be described as conscious, deliberate, explicit, rational and controlled, contrasting with the other (non-analytical reasoning), which is unconscious, associative, implicit, intuitive, and automatic (Stanovich and West, 2000; Shafir and LeBouef, 2002; Kahneman, 2011). A significant research tradition in psychology (Baron, 2007; Kahneman, 2011) demonstrates that heuristics (mental rules of thumb) may be used in the non-analytical mode to reach fast decisions with approximate accuracy; however, heuristics can also produce cognitive bias, resulting in error.

Researchers and theorists have recommended a range of training and intervention options for medical students and doctors based on the dual-process model to enhance their analytical and non-analytical reasoning. Our recent systematic review of dual-process interventions for diagnostic accuracy (Lambe et al., 2016) revealed a burgeoning body of work with some distinctly promising results.

While many studies find some effect of interventions, guided reflection interventions, which encourage a switch from non-analytical to analytical reasoning, emerged as the most consistently successful across five studies (Mamede et al., 2008, 2010b, 2012a; Myung et al., 2013; Schmidt et al., 2014).

While there has been some variation across a number of studies ((Mamede et al., 2008, 2010a,b); (Mamede et al., 2012a,b); (Ilgen et al., 2011, 2013; Myung et al., 2013; Schmidt et al., 2014)) in the specific phrasing and framing of instructions, guided reflection interventions follow a similar structure. Participants are provided with a clinical scenario and asked to diagnose the patient using the following process:

1. Offer the first diagnosis to come to mind and re-examine the information in the clinical scenario.
2. Find details in the scenario that support and refute the hypothesis.
3. Offer alternative possible diagnoses.
4. Find details in the scenario that support and refute each of these hypotheses.
5. Rank the hypotheses in order of likelihood and offer a final diagnosis.

While much of the existing research has focused on the utility of reflective practice as a learning tool (Mamede et al., 2008, 2012b), there is also interest in how these and related metacognitive techniques (which involve awareness and reflection on one's own cognitive processes) may be employed in practice (Croskerry, 2003; Sinclair and Croskerry, 2010; Graber et al., 2012; Croskerry et al., 2013b); that is to say, reflection has been framed as both an educational strategy, to improve future diagnosis, and a workplace strategy, to improve diagnosis in the moment (Croskerry et al., 2013b). A recent landmark review highlights the particular value of reflection at the verification stage of diagnosis, and that reflective interventions that draw the diagnostician's attention directly to the evidence in the case are of most benefit (Mamede and Schmidt, 2017).

Questions remain to be answered before these interventions can be confidently recommended to medical educators and practitioners. There has been limited commentary on the potential of interventions of this sort to have unintended or adverse consequences, or on the trade-offs inherent in promoting one mode of reasoning over another (Norman and Eva, 2003). The aim of this study is to examine two potential pitfalls that may arise in using guided reflection to switch to the analytical style of diagnostic reasoning.

First, there is the concern that adopting reflective methods of diagnosis is time-consuming. Delays in providing a diagnosis may occur due to a more reflective approach to diagnosis in both the short term (longer history-taking, second-guessing by clinicians, bedside consideration) and long term (additional testing, “paralysis by analysis”) (Scott, 2009). These delays have obvious implications for the patient awaiting treatment, and also take time from other patients requiring attention, impacting on the clinician's general efficiency (Berner and Graber, 2008).

Second, there is the concern that specific attempts at debiasing can have deleterious effects on reasoning and compound other biases (Lilienfeld et al., 2009). For example, a clinician attempting to make use of a “consider the alternatives” strategy (Croskerry et al., 2013b) may find that alternative diagnoses do not come easily to mind, leading them to conclude that there are not many good alternatives—and thereby strengthening their confidence that their working diagnosis is correct, by means of the availability bias. This effect has been confirmed in the psychology literature; Sanna et al. (2002) found that participants who were asked to generate many counterfactual alternatives demonstrated increased hindsight bias, compared to participants who were asked to generate only a few.

In light of the concern that reflection is excessively time-consuming, it is important to design the most efficient version of any such diagnostic intervention and to identify the “active ingredients.” This study therefore compares a short version of the guided reflection task with a long version, wherein diagnosticians are asked to generate either two or six alternative diagnoses. If the two versions produce similar effects, it may suggest that diagnostic benefits are possible even with a relatively short reflective process.

In light of the concern that reflection may induce additional bias, the study examines whether diagnosticians who must generate a large number of alternatives become more confident in their initial diagnosis, indicating a hindsight bias, than those who must generate a smaller number of alternatives.

Relatedly, the study also examines what happens when an initial diagnosis is changed as the result of extensive reflection, and compares whether the short or long version of the task is more likely to lead to correction of an incorrect initial diagnosis, or a correct initial diagnosis being discounted in favour of an incorrect diagnosis (which shall be described as “backfiring” for the purpose of this study).

Therefore, the hypotheses under examination are as follows:

1. The accuracy of final diagnoses will differ between participants who are required to generate a greater number of alternative diagnoses and those who are required to generate fewer alternatives, and between both these sets of participants and those who are required to generate no alternatives.
2. Participants who are required to generate a greater number of possible alternative diagnoses will be less likely to lose confidence in their original diagnosis over the course of the guided reflection task than those who are required to generate fewer alternatives.
3. Where a final diagnosis differs from the initial diagnostic hypothesis, rates of correction (change from an incorrect initial diagnosis to a correct final diagnosis) or backfiring (change from a correct initial diagnosis to an incorrect final diagnosis), will differ between participants who are required to generate a greater number of alternative diagnoses and those who are required to generate fewer alternatives.

This study contributes a number of novel points to the literature. First, the study represents an attempt to identify how the guided reflection method may be translated from an extensive educational aid into a practical, concise tool for use in diagnosis. To our knowledge, this is the first empirical study of whether a shorter, less demanding version of the guided reflection method may be as effective as a longer, more thorough version. Second, as outlined above, the potential for unintended consequences or even diagnostic backfiring has been largely neglected in the literature to date; this study attempts to address some of these concerns empirically. Third, the study draws more extensively than most studies to date on perspectives from psychological science and highlights in a more nuanced way the potential for interaction between individual psychological mechanisms (specifically, the interaction between the availability bias and hindsight bias as a potential area of vulnerability for diagnosticians).

## Methods

### Design

An experiment was conducted using a between-groups design. Participants were asked to diagnose four fictional clinical cases, by first impressions (control condition) or by using a short or long guided reflection process. Participants rated their confidence in their initial diagnostic hypothesis at intervals throughout the process. There were two outcomes of interest: (1) change in confidence judgements in the initial diagnostic hypothesis, and (2) accuracy of final diagnosis.

### Participants

Student participants (*n* = 186) were recruited from an undergraduate medical course during their psychiatry rotation. Students were in their fourth (*n* = 101) and fifth (*n* = 85) years of study. Although no gender information was gathered about the students who took part in the study, approximately 58% of students overall in each year group were female. Students had received no specific prior instruction in issues concerning diagnostic reasoning as part of their curriculum.

Power calculations indicated a sample size of 165 (55 per group) for a medium effect size with 0.80 power for ANOVA; this is in line with some larger studies in the literature upon which the present study builds (e.g., Ilgen et al., 2011).

Participants were randomly allocated to control (*n* = 64) or one of two experimental conditions, completing either a short (*n* = 58) or long (*n* = 64) diagnostic table.

### Materials

#### Vignettes

Participants were asked to diagnose a series of four fictional clinical case scenarios. These vignettes are drawn from a bank compiled by Friedman et al., at University of Michigan and have been used in similar research on diagnostic reasoning (Friedman et al., 1999; Payne, 2011). The vignettes are based on real patient cases and represent both common and uncommon or atypical presentations. The case authors provide a definitive correct diagnosis for each case, which is used as the gold standard for participant responses; the correct diagnoses were appendicitis, amoebic liver, colon cancer and Guillaine Barre Syndrome. A small number of additional diagnoses, which represented more specific diagnoses that fitted under the gold standard diagnosis, were also scored as correct. A member of the research team adapted some of the language in the cases for Irish readers.

#### Diagnostic process

For each clinical scenario, participants in the control condition were asked to write down the first diagnosis that comes to mind and to rate their confidence in this diagnosis.

**Figure 1:**
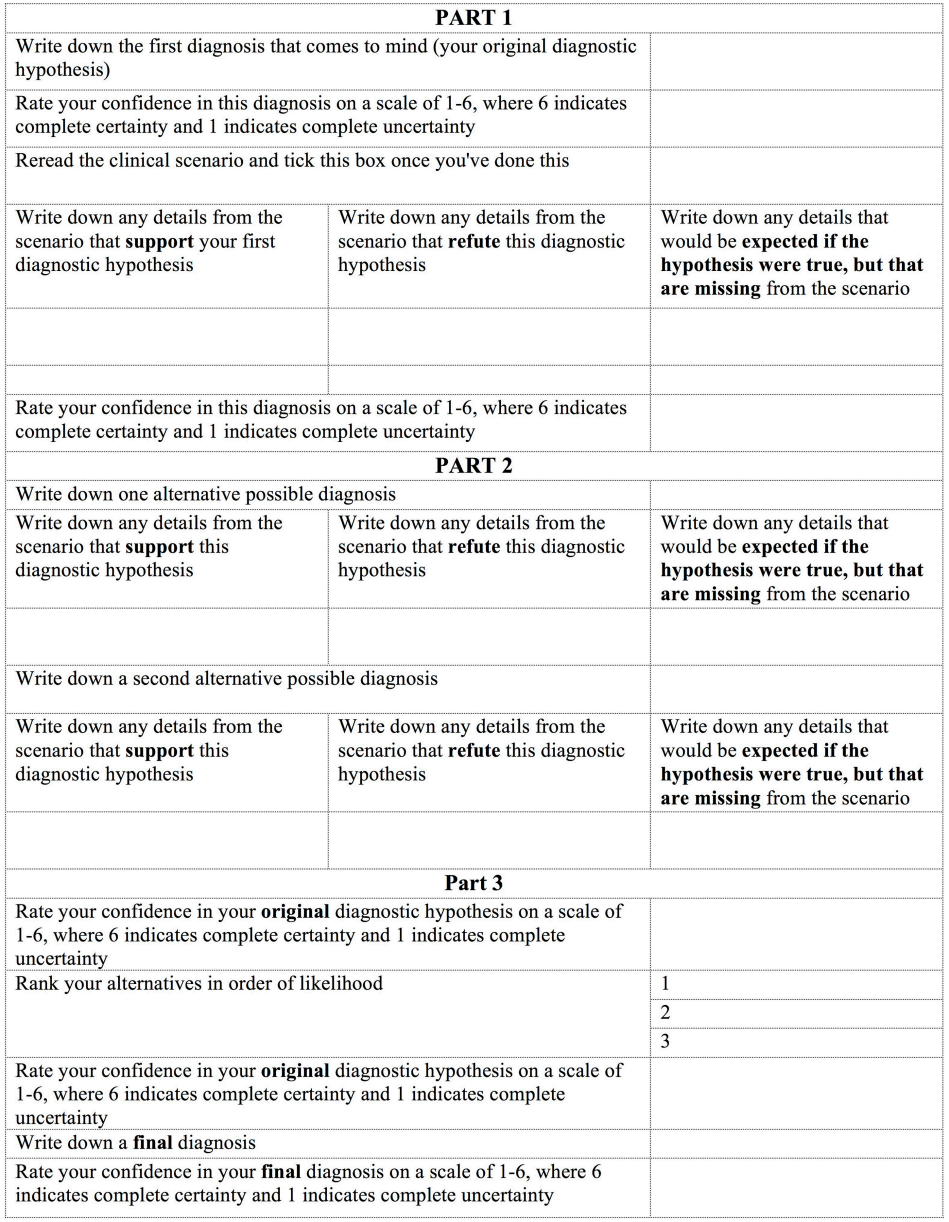
Diagnostic table (“Short” condition).

For each clinical scenario, participants in the experimental conditions were asked to complete a guided reflection table in order to reach a final diagnosis. The table followed the procedure laid out in previous guided reflection studies (Mamede et al., 2008) and is shown in Figure 1.

Participants in the first experimental group (“short”) received a table asking them to generate two possible alternative diagnoses. Participants in the second experimental group (“long”) received a table asking them to generate six possible alternative diagnoses.

### Procedure

Ethical approval was provided by the university's School of Medicine. Recruitment took place during four lecture sessions. Participants were verbally informed about the research by two members of the research team and invited to take part. Booklets containing the vignettes and diagnostic tables were randomly distributed to the students, and the students were instructed to work through the cases in silence. Students were given 60 min to complete the booklet and were advised to spend no more than 15 min on each case; they were notified of the time remaining at 15 min intervals. Following participation, students were debriefed and a short explanation of the key principles of diagnostic error was provided.

### Analysis

Drawing on previous studies of guided reflection interventions (e.g., Mamede et al., 2008), diagnostic accuracy was scored in three ways.

1. Under “first impression” scoring, only the initial diagnostic hypothesis for each case was considered. This diagnosis was counted as being correct only if the participant selected the correct diagnosis as their first *diagnostic hypothesis*.
2. Under strict scoring, a case was counted as having been diagnosed correctly only if the participant selected the correct diagnosis as their *final diagnosis*.
3. Under lenient scoring, a case was counted as having been diagnosed correctly if the participant included the correct diagnosis as one *of their alternatives*, whether or not they ultimately selected this as their final diagnosis.

Change in confidence was calculated by subtracting the participant's confidence rating in their final diagnosis from their confidence rating in their first diagnosis. This comparison was chosen as it represents the most important shift in confidence [confidence at the beginning of the process, where the initial hypothesis has been shown to be particularly powerful (Kostopoulou et al., 2017), and at the end, when action will presumably be taken] and most succinctly reflects the change in confidence over the course of the reflection exercise.

Significance was set at *P* = 0.05 for all tests.

## Results

### Confirmation of manipulation

An independent samples *t*-test confirmed that the mean number of alternatives generated by participants in the long condition (*n* = 58, *M* = 3.03) was significantly higher than the mean number generated by participants in the short condition (*n* = 56, *M* = 1.86), *t*_(61.92)_ = 6.76, *p* < 0.001, two-tailed.

As the number of alternatives generated did not meet parametric assumptions of normality and homogeneity of variance, a Mann-Whitney *U* test was also performed; this also revealed a significant difference in the number of alternatives generated by participants in the short condition (*n* = 56, *Md* = 2.0) and the long condition (*n* = 58, *Md* = 3.0), *U* = 548, *z* = 6.31, *p* < 0.001, *r* = 0.59.

### Diagnostic accuracy

Table 1 presents descriptive statistics for the proportions of cases diagnosed correctly under first impressions, strict scoring and lenient scoring for each of the experimental conditions.

**Table 1:** Mean diagnostic accuracy scores and standard deviations.

**Condition**	**First impression**	**Strict (correct final diagnosis)**	**Lenient (correct diagnosis included in alternatives)**
	**M (SD)**	**M (SD)**	**M (SD)**
Control (*n* = 64)	0.47 (0.26)	0.47 (0.26)^*^	0.47 (0.26)^*^
Short (*n* = 55)	0.49 (0.24)	0.42 (0.24)	0.70 (0.23)
Long (*n* = 57)	0.45 (0.24)	0.41 (0.26)	0.72 (0.25)

* *Under the control condition, the first impression diagnosis also constitutes the final diagnosis and no alternative diagnoses are offered. Therefore, the scores for the control condition under first impression, strict scoring and lenient scoring are all equal*.

A one-way between groups analysis of variance revealed no significant differences in the accuracy of final diagnosis (strict scoring) between any of the three conditions, *F*_(2, 173)_ = 0.841, *p* > 0.05.

A one-way between groups analysis of variance revealed a significant difference in accuracy of diagnostic alternatives (lenient scoring) between the three conditions: *F*_(2, 175)_ = 18.992, *p* < 0.001. The effect size, calculated using eta squared, was 0.18. *Post-hoc* comparisons using the Tukey HSD test indicated that the mean scores for the short and long conditions (*M* = 0.70, *SD* = 0.23 and *M* = 0.72, *SD* = 25, respectively) did not differ significantly from one another, but were significantly higher than the control condition (*M* = 0.47, *SD* = 0.26).

### Change in confidence

An independent samples *t*-test revealed no significant difference in change in confidence between the short (*M* = 0.11, *SD* = 0.59) and long (*M* = 0.10, *SD* = 0.61) groups: *t*_(109)_ = 0.52, *p* > 0.05.

A one-way between groups analysis of variance revealed a significant difference in confidence before the reflective process between the three conditions: *F*_(2, 176)_ = 3.48, *p* < 0.05. *Post-hoc* comparisons using the Tukey HSD test indicated that confidence was significantly higher in the long condition (*M* = 3.15, *SD* = 0.80) than in the control condition (*M* = 2.79, *SD* = 0.77). No differences in confidence were observed at the end of the reflective process.

### Rates of correction and backfiring

Across the short and long form groups, participants changed their final diagnosis from their initial diagnostic hypothesis in 17% of cases (*n* = 70 cases). Table 2 presents the contingency table for diagnoses changed from incorrect to correct (corrected) and diagnoses changed from correct to incorrect (backfired) in the short and long form conditions.

**Table 2:** Contingency table for changed diagnoses in short and long conditions (*n* = 70 cases).

**Condition**	**Diagnoses changed from incorrect to correct (corrected)**	**Diagnoses changed from correct to incorrect (backfired)**
	***n* (%)**	***n* (%)**
Short	16 (38)	26 (62)
Long	14 (50)	14 (50)
Total	30 (43)	40 (57)

A Chi-square test for independence indicated no significant association between group and correction/backfiring of diagnosis, χ2 (1, *n* = 70) = 0.972, *p* > 0.05, *phi* = 0.118.

## Discussion

### Diagnostic accuracy

Results showed that neither participants using the short nor long version of the guided reflection intervention were more accurate in their final diagnoses than participants who diagnosed based on their first impressions only. This is in contrast with some previous studies that found a beneficial effect for diagnosis, particularly of complex cases (Mamede et al., 2008, 2010b; Ilgen et al., 2011; Myung et al., 2013).

Two factors may explain these results. First, the lack of improvement with reflection may arise due to the reasoning strategies employed by medical students in particular. Although these students had not received prior instruction in any particular style of diagnostic reasoning, there is some evidence that novice diagnosticians rely more on analytical reasoning processes than experts (Kulatunga-Moruzi et al., 2001). If this is the case, participants in this study may have offered a “first impression” diagnosis that was in fact the product of an analytical reasoning style that more closely resembles the guided reflection model than the non-analytical, mode of reasoning we intended to elicit by requesting a “first impressions” diagnosis. Future studies may benefit from methodological adjustments to parse these effects, for example restricted time limits for non-analytical “control” conditions or think-aloud protocols.

Second, although the cases were clearly difficult, given the low accuracy scores overall, they were not structurally complex; no secondary diagnosis was present in any case. Previous studies indicate a particular benefit an effect of reflective practice on accuracy of final diagnosis for complex cases only (Mamede et al., 2008). This may explain the absence of an effect on accuracy of final diagnoses.

Results showed that the guided reflection intervention was associated with the generation of more accurate hypotheses than diagnosis based on first impressions only. Statistically speaking, this finding is unsurprising, as it is logical to expect a correct diagnosis to be named with three or seven “chances” compared to just one.

However, it was also shown that participants who were asked to generate two alternative diagnostic hypotheses provided an accurate diagnostic hypothesis as frequently as those who were asked to generate six alternatives. In this way, instructions to generate many alternatives did not increase the accuracy of alternatives, suggesting that a relatively short version of the guided reflection process may yield similar accuracy rates as a longer, more burdensome version.

As mentioned above, the “lenient” scoring logically allows participants to perform better in the experimental conditions than in the control condition simply through chance. However, by considering the accuracy of the set of alternatives as a whole, lenient scoring arguably has more ecological validity for the clinical setting, where doctors typically consider (however briefly) more than one diagnosis. This finding underscores the importance of the differential diagnosis and the need to avoid premature closure; that is, the tendency to call off a search for the correct or complete diagnosis once an adequate diagnosis has been identified (Graber et al., 2005).

Relatedly, the choice between focusing on the accuracy of a final diagnosis vs. the accuracy of alternatives highlights the assumption that there exists for each case a single correct diagnosis. For this study, we scored accuracy for a “gold standard” answer along with a small range of other acceptable answers. Defining error and accuracy is important for both experimental studies going forward and for the field at large.

Previous studies of the guided reflection method have not instructed participants to generate a particular number of alternative diagnoses for test cases, and these findings suggest that the number of diagnostic alternatives generated may not be a crucial component of the success of guided reflection interventions. The metacognitive processes induced by the instruction to generate alternatives and the evidence-gathering process to investigate those alternatives may be of more value; that is, the quality of reasoning processes may contribute more than the exhaustiveness or extent of reasoning to diagnostic success. This is in line with existing research; while “consider the alternatives” is a commonly suggested strategy, it has seldom been examined empirically and the evidence that it is, in itself, sufficient to improve accuracy is lacking (Regehr et al., 1994; Feyzi-Behnagh et al., 2014; Lambe et al., 2016). Specific reasoning instructions that draw attention to the evidence in the case have been found to be substantially more effective than more general metacognitive instructions to take care and consider all the data (Mamede and Schmidt, 2017). Not all reflective interventions are alike, and not all studies find evidence for their effectiveness; it is therefore important to identify the “active ingredient” in the guided reflection process to ensure that the most efficient version of the task may be recommended to diagnosticians and medical educators. These findings suggest that the optimal version of a guided reflection intervention may include instructions to generate only a few alternatives.

It is possible that the generation of additional alternatives offered no additional benefit to diagnostic accuracy due to the additional cognitive load required by a more extensive differential diagnosis (Payne, 2011). This may be particularly the case for the novice diagnosticians who took part in this study; even with a pen-and-paper booklet to assist them, they may have struggled to consider more than two or three alternatives at a time. Indeed, even when asked to offer six alternatives, participants offered an average of only three.

### Diagnostic confidence

Participants who completed a longer version of the task exhibited a similar change in confidence as those who completed the shorter version of the task; that is, the change in confidence across both groups was minimal. This study therefore finds no support for concerns raised in the literature (Lilienfeld et al., 2009) that an availability bias induced by such a reflective exercise may compromise diagnostic accuracy, nor does it find evidence that generating alternatives bolsters or diminishes diagnostic confidence among this participant group.

Again, the natural use of analytical reasoning processes (despite no formal training in these) may explain the minimal change in confidence throughout the task; if the “first impression” diagnosis listed is in fact the product of more careful consideration, fluctuations in confidence would have preceded the written task and therefore not have been captured. Additionally, given the similar rates of correction and backfiring, it is possible that medical students' confidence is simply not well calibrated; they do not know when they are wrong and may not yet have developed good strategies for actively assessing their own diagnoses.

### Correction and backfiring

Where a final diagnosis differed from the initial diagnostic hypothesis, participants who were required to generate a greater number of alternatives were no more likely to correct an incorrect initial diagnostic hypothesis than those who were required to generate fewer alternatives.

Two phenomena are of interest here. First, participants seldom changed their minds about their initial diagnosis; in 83% of cases, the original diagnostic hypothesis was retained as the final diagnosis. Second, participants were as likely to change from an incorrect to a correct diagnosis as they were to change from a correct to an incorrect diagnosis. This suggests that, for this participant group, the utility of the guided reflection exercise in catching errors is relatively limited, and opens up inexperienced diagnosticians to the risk of “talking themselves out of” a correct diagnosis.

These findings, around diagnostic accuracy generally and rates of correction and backfiring specifically, highlight the importance of the first impression in diagnosis. Participants were able to identify accurate alternatives to their initial diagnostic hypothesis, but seldom actually changed their minds from their first impression. This reflects existing research; studies have previously found a strong association between the first diagnostic impression and subsequent diagnostic and treatment choices (Kostopoulou et al., 2017), and doctors tend to be highly confident in their initial diagnosis (Monteiro et al., 2015). While this potent effect of the first impression may leave doctors vulnerable to certain cognitive pitfalls, such as an anchoring bias or confirmation bias (Croskerry et al., 2013a), the results of this study suggest that the novice diagnostician's initial diagnosis is as likely to be correct as a final diagnosis chosen after a relatively lengthy reflective exercise. As such, reflection strategies do not reliably help the novice to choose the correct final diagnosis, even when they identify the correct answer as a possibility.

If it is the case, as these results suggest, that reflective exercises seldom lead a doctor to deviate from their first impression, non-analytical reasoning may be the primary driver of many diagnostic decisions. The large majority of interventions based on the dual process model to improve diagnostic accuracy target analytical reasoning (Lambe et al., 2016), with little focus on improving non-analytical reasoning. This is perhaps unsurprising; accessing and manipulating non-analytical processes is difficult, due to their very nature as unconscious mechanisms. However, improvements in how trainees build up their non-analytical and pattern recognition skills are possible through changes in medical education curricula and emerging computer and simulation technologies. These are exciting new possibilities for research in this area.

### Limitations

A number of limitations should be acknowledged in the interpretation of these findings. First, participants were senior medical students, and not expert diagnosticians; as reliance on intuitive reasoning in diagnosis may increase over the course of a doctor's career (Ilgen et al., 2011), these findings may not be generalizable to doctors with more expertise. Second, given the relatively low accuracy scores across all conditions, the cases used in this study may have been too challenging for this participant group, which may have introduced a ceiling effect. Third, participants in the short and long conditions had equal amounts of time to complete the task. It is possible that participants in the short condition spent additional time on each case, or that participants in the long condition did not have sufficient time to complete the task in full for each case. This arose due to the group classroom setting of the data collection sessions but should be avoided for future studies; it seems reasonable that more reflective reasoning processes will take place if one is allowed much time to think about a “first” diagnostic impression. Fourth, participants in the long condition on average provided only three out of the required six alternatives. Although this is a statistically larger number of alternatives than provided by participants in the short condition, it is worth considering why participants provided fewer alternatives than required; it is possible that the cases were too clear-cut to generate a large number of reasonable alternatives, or that the time allowed was not sufficient to carry out a full analysis of each alternative. These issues may be corrected with future studies. Fifth, as with the majority of studies in this area, the use of fictional vignettes and the laboratory environment in which this study was conducted limits the generalisability of findings to a real clinical setting. Sixth, in considering the potential for extensive reflection to “backfire,” we only consider cognitive factors, such as availability bias, in the somewhat artificial context of all information being available to the diagnostician. The study does not allow us to determine the precise process by which participants change their mind about a diagnosis, only to observe the frequency with which this occurs. Non-cognitive issues, such as additional tests leading to inflated risk of false positives, overtesting, and overdiagnosis, are also enormously significant factors in potential backfiring (Scott, 2009). These factors are beyond the scope of this study but provide fertile ground for future studies.

An additional limitation reflects a potential confound in the study (and some others in the literature upon which the methodology was modelled); participants in the control condition only had the opportunity to list a single hypothesis, when in reality it is possible that these participants may have considered more than one diagnosis very rapidly from the early stages of each vignette. This study is unable to determine whether the greater accuracy of the alternatives generated by participants in the short and long conditions is an effect of the reflective intervention or merely an artefact of the restrictions placed on participants in the control condition. As such, we suggest that future research includes a condition in which participants offer their first impressions without restrictions or imposed structure, as soon as it comes to mind; comparison of accuracy in this condition compared with accuracy in the reflective condition(s) will help to elucidate the intervention's effect and eliminate the present confound.

### Conclusions

This study finds no evidence to support the use of the guided reflection method as a diagnostic aid for novice diagnosticians using standard methods in the literature. While our findings suggest that a shorter, less demanding version of the process can elicit accurate diagnostic hypotheses at a similar rate as a longer version, the accuracy of final diagnosis was ultimately not improved by any version of the reflection task with this participant group. The findings also highlight the durability of an initial diagnosis and the relative inertia of diagnostic decisions.

Future studies should continue to test alternative versions of the task to identify those factors that are most effective in improving accuracy in order to create the most efficient possible intervention. Identifying cases and situations in which reflection may offer the greatest benefit should also be a priority for this research area. It is also important that such studies examine the generalisability of such efforts to trainees and practitioners at all levels; understanding development differences in diagnostic reasoning may provide important insights into the mechanisms by which diagnostic expertise develops, as well as, point to the most appropriate interventions for reducing error and enhancing reasoning at every stage of a doctor's career. Finally, methodological improvements to parse the effect of the intervention from other factors in experimental studies and to eliminate potential confounds, as outlined above, will be important in clarifying and strengthening the case for continued development of guided reflection interventions.

## Ethics statement

This study was carried out in accordance with the recommendations of the Trinity College Guidelines for Good Research Practice and the Trinity College Dublin School of Medicine Research Ethics Committee.

The study did not require any identifying information to be collected. As such, the study took the form of an anonymous survey, the completion of which implied consent. Participants were provided with a participant information leaflet and were invited to ask any questions before completing the assessment.

The protocol was approved by the Trinity College Dublin School of Medicine Research Ethics Committee.

## Author contributions

KAL designed the study, sourced stimulus materials, carried out data collection and analysis, and drafted and revised the paper. DH co-designed the study, supervised data analysis, and critically revised the paper. BDK co-designed the study, reviewed stimulus materials, facilitated data collection, and critically revised the paper.

### Conflict of interest statement

The authors declare that the research was conducted in the absence of any commercial or financial relationships that could be construed as a potential conflict of interest.
